# Supplementary material for: HIVprotI: an integrated web based platform for prediction and design of HIV proteins inhibitors
Source: J Cheminform. 2018 Mar 9;10:12. doi: 10.1186/s13321-018-0266-y (PMC5845081; doi:10.1186/s13321-018-0266-y)
Supplement: Supplementary file 2 — Additional file 2. Source code of HIVProtI web server. [file 13321_2018_266_MOESM2_ESM.zip › HIVprotI_Source-code/hivprotI/weka-3-6-6/doc/weka/core/neighboursearch/LinearNNSearch.html]

LinearNNSearch


---


|  |  |  |
| --- | --- | --- |
| **Overview** \| **Package** \| **Class** \| **Tree** \| **Deprecated** \| **Index** \| **Help** \| **Weka's home** | |  |
| **PREV CLASS**   **NEXT CLASS** | **FRAMES**    **NO FRAMES**     **All Classes** |  |
| SUMMARY: NESTED \| FIELD \| CONSTR \| METHOD | DETAIL: FIELD \| CONSTR \| METHOD |  |


---


## weka.core.neighboursearch Class LinearNNSearch

```
java.lang.Object
  weka.core.neighboursearch.NearestNeighbourSearch
      weka.core.neighboursearch.LinearNNSearch
```

**All Implemented Interfaces:**: java.io.Serializable, AdditionalMeasureProducer, OptionHandler, RevisionHandler

---

``` public class LinearNNSearch extends NearestNeighbourSearch ```

Class implementing the brute force search algorithm for nearest neighbour search.


Valid options are:

```
 -S
  Skip identical instances (distances equal to zero).
```

**Version:**
:   $Revision: 1.2 $

**Author:**
:   Ashraf M. Kibriya (amk14[at-the-rate]cs[dot]waikato[dot]ac[dot]nz)

**See Also:**: Serialized Form

---

**Constructor Summary**

`LinearNNSearch()`
  
          Constructor.

`LinearNNSearch(Instances insts)`
  
          Constructor that uses the supplied set of
instances.


| **Method Summary** | |
| --- | --- |
| `void` | `addInstanceInfo(Instance ins)`             Adds the given instance info. |
| `double[]` | `getDistances()`             Returns the distances of the k nearest neighbours. |
| `java.lang.String[]` | `getOptions()`             Gets the current settings. |
| `java.lang.String` | `getRevision()`             Returns the revision string. |
| `boolean` | `getSkipIdentical()`             Gets whether if identical instances are skipped from the neighbourhood. |
| `java.lang.String` | `globalInfo()`             Returns a string describing this nearest neighbour search algorithm. |
| `Instances` | `kNearestNeighbours(Instance target, int kNN)`             Returns k nearest instances in the current neighbourhood to the supplied instance. |
| `java.util.Enumeration` | `listOptions()`             Returns an enumeration describing the available options. |
| `Instance` | `nearestNeighbour(Instance target)`             Returns the nearest instance in the current neighbourhood to the supplied instance. |
| `void` | `setInstances(Instances insts)`             Sets the instances comprising the current neighbourhood. |
| `void` | `setOptions(java.lang.String[] options)`             Parses a given list of options. |
| `void` | `setSkipIdentical(boolean skip)`             Sets the property to skip identical instances (with distance zero from the target) from the set of neighbours returned. |
| `java.lang.String` | `skipIdenticalTipText()`             Returns the tip text for this property. |
| `void` | `update(Instance ins)`             Updates the LinearNNSearch to cater for the new added instance. |

**Methods inherited from class weka.core.neighboursearch.NearestNeighbourSearch**

`combSort11, distanceFunctionTipText, enumerateMeasures, getDistanceFunction, getInstances, getMeasure, getMeasurePerformance, getPerformanceStats, measurePerformanceTipText, quickSort, setDistanceFunction, setMeasurePerformance`

**Methods inherited from class java.lang.Object**

`equals, getClass, hashCode, notify, notifyAll, toString, wait, wait, wait`

**Constructor Detail**

### LinearNNSearch

```
public LinearNNSearch()
```

:   Constructor. Needs setInstances(Instances)
    to be called before the class is usable.

---


### LinearNNSearch

```
public LinearNNSearch(Instances insts)
```

:   Constructor that uses the supplied set of
    instances.

    **Parameters:**: `insts` - the instances to use


**Method Detail**

### globalInfo

```
public java.lang.String globalInfo()
```

:   Returns a string describing this nearest neighbour search algorithm.

    :   **Overrides:**: `globalInfo` in class `NearestNeighbourSearch`
    :   **Returns:**: a description of the algorithm for displaying in the explorer/experimenter gui

---


### listOptions

```
public java.util.Enumeration listOptions()
```

:   Returns an enumeration describing the available options.

    :   **Specified by:**: `listOptions` in interface `OptionHandler` **Overrides:**: `listOptions` in class `NearestNeighbourSearch`
    :   **Returns:**: an enumeration of all the available options.

---


### setOptions

```
public void setOptions(java.lang.String[] options)
                throws java.lang.Exception
```

:   Parses a given list of options.


    Valid options are:

    ```
     -S
      Skip identical instances (distances equal to zero).
    ```

    :   **Specified by:**: `setOptions` in interface `OptionHandler` **Overrides:**: `setOptions` in class `NearestNeighbourSearch`
    :   **Parameters:**: `options` - the list of options as an array of strings **Throws:**: `java.lang.Exception` - if an option is not supported

---


### getOptions

```
public java.lang.String[] getOptions()
```

:   Gets the current settings.

    :   **Specified by:**: `getOptions` in interface `OptionHandler` **Overrides:**: `getOptions` in class `NearestNeighbourSearch`
    :   **Returns:**: an array of strings suitable for passing to setOptions()

---


### skipIdenticalTipText

```
public java.lang.String skipIdenticalTipText()
```

:   Returns the tip text for this property.

    :   **Returns:**: tip text for this property suitable for displaying in the explorer/experimenter gui

---


### setSkipIdentical

```
public void setSkipIdentical(boolean skip)
```

:   Sets the property to skip identical instances (with distance zero from
    the target) from the set of neighbours returned.

    :   **Parameters:**: `skip` - if true, identical intances are skipped

---


### getSkipIdentical

```
public boolean getSkipIdentical()
```

:   Gets whether if identical instances are skipped from the neighbourhood.

    :   **Returns:**: true if identical instances are skipped

---


### nearestNeighbour

```
public Instance nearestNeighbour(Instance target)
                          throws java.lang.Exception
```

:   Returns the nearest instance in the current neighbourhood to the supplied
    instance.

    :   **Specified by:**: `nearestNeighbour` in class `NearestNeighbourSearch`
    :   **Parameters:**: `target` - The instance to find the nearest neighbour for. **Returns:**: the nearest instance **Throws:**: `java.lang.Exception` - if the nearest neighbour could not be found.

---


### kNearestNeighbours

```
public Instances kNearestNeighbours(Instance target,
                                    int kNN)
                             throws java.lang.Exception
```

:   Returns k nearest instances in the current neighbourhood to the supplied
    instance.

    :   **Specified by:**: `kNearestNeighbours` in class `NearestNeighbourSearch`
    :   **Parameters:**: `target` - The instance to find the k nearest neighbours for.: `kNN` - The number of nearest neighbours to find. **Returns:**: the k nearest neighbors **Throws:**: `java.lang.Exception` - if the neighbours could not be found.

---


### getDistances

```
public double[] getDistances()
                      throws java.lang.Exception
```

:   Returns the distances of the k nearest neighbours. The kNearestNeighbours
    or nearestNeighbour must always be called before calling this function. If
    this function is called before calling either the kNearestNeighbours or
    the nearestNeighbour, then it throws an exception. If, however, if either
    of the nearestNeighbour functions are called at any point in the
    past then no exception is thrown and the distances of the training set from
    the last supplied target instance (to either one of the nearestNeighbour
    functions) is/are returned.

    :   **Specified by:**: `getDistances` in class `NearestNeighbourSearch`
    :   **Returns:**: array containing the distances of the nearestNeighbours. The length and ordering of the array is the same as that of the instances returned by nearestNeighbour functions. **Throws:**: `java.lang.Exception` - if called before calling kNearestNeighbours or nearestNeighbours.

---


### setInstances

```
public void setInstances(Instances insts)
                  throws java.lang.Exception
```

:   Sets the instances comprising the current neighbourhood.

    :   **Overrides:**: `setInstances` in class `NearestNeighbourSearch`
    :   **Parameters:**: `insts` - The set of instances on which the nearest neighbour search is carried out. Usually this set is the training set. **Throws:**: `java.lang.Exception` - if setting of instances fails

---


### update

```
public void update(Instance ins)
            throws java.lang.Exception
```

:   Updates the LinearNNSearch to cater for the new added instance. This
    implementation only updates the ranges of the DistanceFunction class,
    since our set of instances is passed by reference and should already have
    the newly added instance.

    :   **Specified by:**: `update` in class `NearestNeighbourSearch`
    :   **Parameters:**: `ins` - The instance to add. Usually this is the instance that is added to our neighbourhood i.e. the training instances. **Throws:**: `java.lang.Exception` - if the given instances are null

---


### addInstanceInfo

```
public void addInstanceInfo(Instance ins)
```

:   Adds the given instance info. This implementation updates the range
    datastructures of the DistanceFunction class.

    :   **Overrides:**: `addInstanceInfo` in class `NearestNeighbourSearch`
    :   **Parameters:**: `ins` - The instance to add the information of. Usually this is the test instance supplied to update the range of attributes in the distance function.

---


### getRevision

```
public java.lang.String getRevision()
```

:   Returns the revision string.

    :   **Returns:**: the revision


---


|  |  |  |
| --- | --- | --- |
| **Overview** \| **Package** \| **Class** \| **Tree** \| **Deprecated** \| **Index** \| **Help** \| **Weka's home** | |  |
| **PREV CLASS**   **NEXT CLASS** | **FRAMES**    **NO FRAMES**     **All Classes** |  |
| SUMMARY: NESTED \| FIELD \| CONSTR \| METHOD | DETAIL: FIELD \| CONSTR \| METHOD |  |


---
